# Supplementary material for: Full-color persistent room temperature phosphorescent elastomers with robust optical properties
Source: Nat Commun. 2023 Aug 10;14:4839. doi: 10.1038/s41467-023-40193-1 (PMC10415293; doi:10.1038/s41467-023-40193-1)
Supplement: Supplementary file 1 — Supplementary Information [file 41467_2023_40193_MOESM1_ESM.pdf]

Supplementary Information

Supplementary Methods

Experimental Procedures

Materials. Unless otherwise stated, all starting materials and reagents were purchased from commercial suppliers and used without further purification. All solvents were purified before use. The solvents were carefully dried and distilled from appropriate drying agents prior to use.

Synthesis and characterization

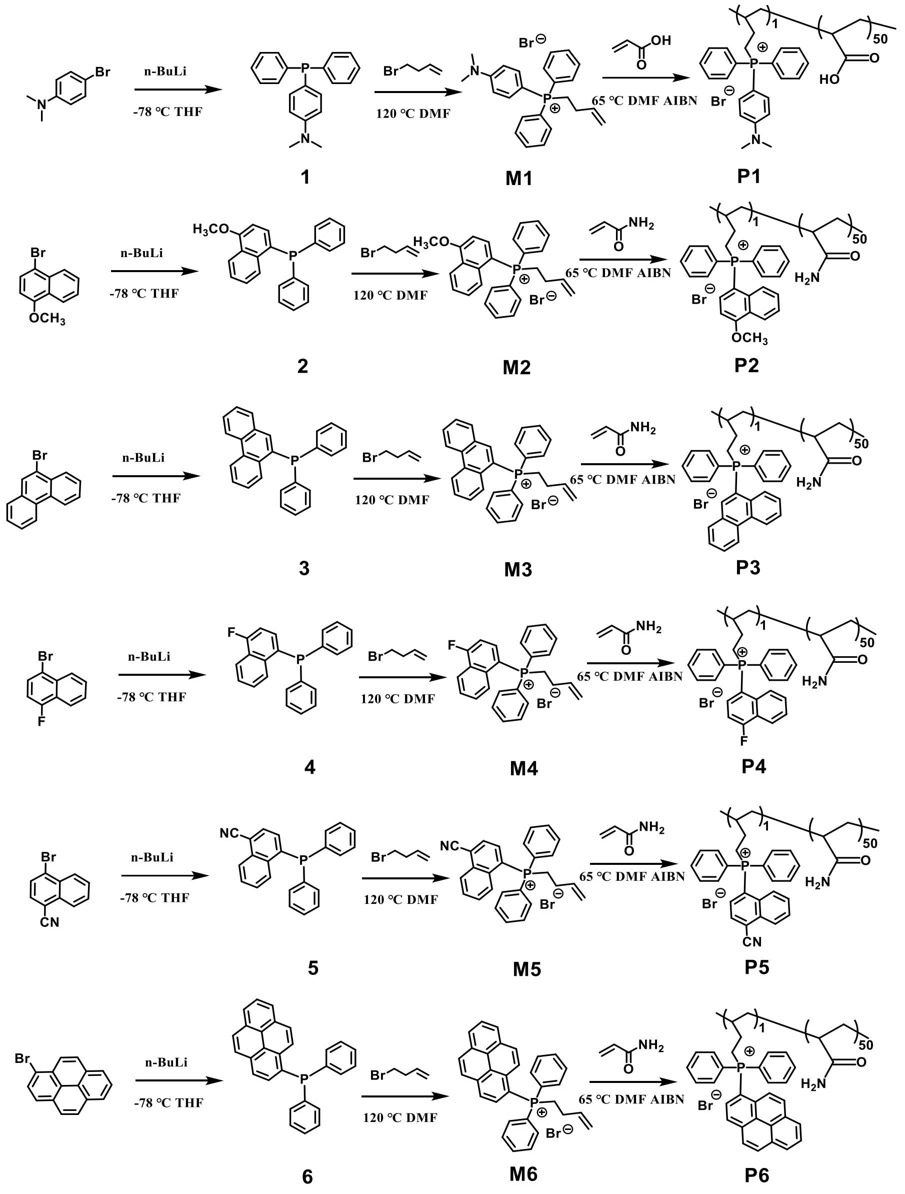

Supplementary Fig. 1 Synthetic route of M1-M6 and P1-P6.

Synthesis of M1:

The 1.6 M hexane solution of n-BuLi (18.7 mL, 30.0 mmol) was added dropwise to an agitating superdry THF solution (40 mL) of 4-Bromo-N,N-dimethylaniline (5.0 g, 25.0 mmol) at -78 °C under an inert atmosphere of nitrogen. After stirring for 1 h, chlorodiphenylphosphine (6.6 g, 30.0 mmol) was added into the mixture. The reaction was stirred at 25 °C for 12 h. 1 was purified through column chromatography (petroleum ether), yield 81%. In nitrogen atmosphere, compound 1 (2 g, 5.55 mmol) was added into the mixture of 4-Bromo-1-butene (2.4 g, 11.1 mmol).and DMF (50 mL). The reaction was stirred at 120 °C for 24 h. M1 was purified through column chroma-tography (methanol : dichloromethane = 50 : 1), yield 89%. ^{1}H NMR (400 MHz, DMSO-d_6, δ): 7.90-7.68 (m, 11H), 7.52-7.40 (m, 2H), 7.08-6.83 (m, 2H), 5.98-5.67 (m, 1H), 5.42-5.02 (m, 2H), 3.73-3.39 (m, 2H), 3.05 (s, 6H), 2.34-2.24 (m, 1H). ^{13}C NMR (100 MHz, DMSO-d_6, δ): 154.14, 136.60, 135.27, 134.63, 133.85, 130.57, 120.96, 120.14, 117.04, 112.79, 99.06, 26.10, 20.38, 18.36. HRMS: m/z = 360.1817 [M − Br].

Synthesis of M2:

The 1.6 M hexane solution of n-BuLi (15.8 mL, 25.3 mmol) was added dropwise to an agitating superdry THF solution (40 mL) of 1-Bromo-4-methoxynaphthalene (5.0 g, 21.1 mmol) at -78 °C under an inert atmosphere of nitrogen. After stirring for 1 h, chlorodiphenylphosphine (4.6 g, 21.09 mmol) was added into the mixture. The reaction was stirred at 25 °C for 12 h. 2 was purified through column chromatography (petroleum ether), yield 76%. In nitrogen atmosphere, 2 (2 g, 5.85 mmol) was added into the mixture of 4-bromo-1-butene (2.5 g, 11.7 mmol) and DMF (20 mL). The reaction was stirred at 120 °C for 24 h. The M2 was purified through column chromatography (methanol : dichloromethane = 50 : 1), yield 83%. ^{1}H NMR (400 MHz, $CDCl_3$, δ): 8.52-8.21 (m, 2H), 7.73 (m, J = 92.9, 50.6, 45.3 Hz, 16H), 6.12-5.90 (m, 1H), 5.04-4.84 (m, 2H), 4.16 (s, 3H), 3.94-3.63 (m, 2H), 2.45-2.30 (m, 2H). ^{13}C NMR (100 MHz, DMSO-d_6, δ): 160.35, 137.15, 136.18, 136.08, 133.63, 131.15, 130.14, 128.71, 127.34, 120.55, 119.51, 118.13, 112.23, 111.56, 106.34, 56.69, 23.58, 23.12, 13.30. HRMS: m/z = 396.1691 [M − Br].

Synthesis of M3:

The 1.6 M hexane solution of n-BuLi (14.5 mL, 23.3 mmol) was added dropwise to an agitating superdry THF solution (40 mL) of 9-Bromophenanthrene (5 g, 19.45 mmol) at -78 °C under an inert atmosphere of nitrogen. After stirring for 1 h, chlorodiphenylphosphine (5.1 g, 23.3 mmol) was added into the mixture. The reaction was stirred at 25 °C for 12 h. 3 was purified through column chromatography (petroleum ether), yield 78%. In nitrogen atmosphere, 3 (2 g, 5.5 mmol) was added into the mixture of 4-bromo-1-butene (1.5 g, 11.0 mmol) and DMF (20 mL). The reaction was stirred at 120 °C for 24 h. The M3 was

purified through column chromatography (methanol : dichloromethane = 50 : 1), yield 87%. ^{1}H NMR (400 MHz, $CDCl_3$, δ): 7.90-7.68 (m, 11H), 7.52-7.40 (m, 2H), 7.08-6.83 (m, 2H), 5.98-5.67 (m, 1H), 5.42-5.02 (m, 2H), 3.73-3.39 (m, 2H), 3.05 (s, 6H), 2.34-2.24 (m, 1H).^{13}C NMR (100 MHz, DMSO-d_6, δ): 140.35, 136.26, 134.48, 133.81, 135.04, 132.85, 132.15, 130.96, 128.09, 125.49 124.25, 121.14, 118.93, 117.27, 112.95, 18.49. HRMS: m/z = 417.1751 [M − Br].

Synthesis of M4:

The 1.6 M hexane solution of n-BuLi (16.7 mL, 26.7 mmol) was added dropwise to an agitating superdry THF solution (40 mL) of 1-bromo-4-fluoronaphthalene (5 g, 22.2 mmol) at -78 °C under an inert atmosphere of nitrogen. After stirring for 1 h, chlorodiphenylphosphine (5.9 g, 26.7 mmol) was added into the mixture. The reaction was stirred at 25 °C for 12 h. 3 was purified through column chromatography (petroleum ether), yield 74%. In nitrogen atmosphere, 4 (2 g, 6.0 mmol) was added into the mixture of 4-bromo-1-butene (1.6 g, 12.1 mmol) and DMF (20 mL). The reaction was stirred at 120 °C for 24 h. The M4 was purified through column chromatography (methanol : dichloromethane = 50 : 1), yield 80%. ^{1}H NMR (400 MHz, $CDCl_3$, δ): 8.36-8.28 (m, 1H), 7.99-7.57 (m, 17H), 5.89-5.58 (m, 1H), 5.29-4.95 (m, 1H), 4.74-4.57 (m, 1H), 4.04-3.73 (m, 1H), 2.48-2.10 (m, 1H). ^{13}C NMR (100 MHz, DMSO-d_6, δ): 167.34 137.29, 164.89, 136.93, 117.73, 115.0, 114.02, 23.67, 20.74, 13.60. HRMS: m/z = 385.1504 [M − Br].

Synthesis of M5:

The 1.6 M hexane solution of n-BuLi (16.1 mL, 25.8 mmol) was added dropwise to an agitating superdry THF solution (40 mL) of 4-bromonaphthalene-1-carbonitrile (5 g, 21.5 mmol) at -78 °C under an inert atmosphere of nitrogen. After stirring for 1 h, chlorodiphenylphosphine (5.5 g, 25.8 mmol) was added into the mixture. The reaction was stirred at 25 °C for 12 h. 5 was purified through column chromatography (petroleum ether), yield 71%. In nitrogen atmosphere, 5 (2 g, 5.9 mmol) was added into the mixture of 4-bromo-1-butene (1.6 g, 11.8 mmol) and DMF (20 mL). The reaction was stirred at 120 °C for 24 h. The M5 was purified through column chromatography (methanol : dichloromethane = 50 : 1), yield 75%. ^{1}H NMR (400 MHz, DMSO-d_6, δ): 8.36-8.28 (m, 1H), 7.99-7.57 (m, 17H), 5.89-5.58 (m, 1H), 5.29-4.95 (m, 1H), 4.74-4.57 (m, 1H), 4.04-3.73 (m, 1H), 2.48-2.10 (m, 1H). ^{13}C NMR (100 MHz, DMSO-d_6, δ): 137.55, 135.73, 135.16, 133.17, 127.19, 120.78, 118.73, 116.65, 27.21, 21.64, 18.23, 13.35. HRMS: m/z = 392.1564 [M − Br].

Synthesis of M6:

The 1.6 M hexane solution of n-BuLi (13.3 mL, 21.34 mmol) was added dropwise to an agitating superdry THF solution (40 mL) of 1-bromopyrene (5 g, 17.78 mmol) at -78 °C under an inert atmosphere of nitrogen. After stirring for 1 h, chlorodiphenylphosphine (3.9 g, 17.78 mmol) was added into the mixture. The reaction was stirred at 25 °C for 12 h. 6 was purified through column chromatography (petroleum ether), yield 65%. In nitrogen atmosphere, 6 (2 g, 5.18 mmol) was added into the mixture of 4-bromo-1-butene (0.8 g, 6.22 mmol) and DMF (20 mL). The reaction was stirred at 120 °C for 24 h. The M6 was purified through column chromatography (methanol : dichloromethane = 50 : 1), yield 50%. ^{1}H NMR (400 MHz, $CDCl_3$): δ 8.46-8.33 (m, 5H), 8.22-8.15 (m, 3H), 8.00-7.94 (m, 3H), 7.69 (d, 3.3 Hz, 8H), 6.16-5.85 (m, 1H), 5.01-4.78 (m, 2H), 4.20-4.15 (m, 1H), 2.53-2.45 (m, 1H), 1.97-1.87 (m, 2H).^{13}C NMR (100 MHz, $CDCl_3$, δ): 136.17, 134.98, 133.79, 133.59, 132.91, 130.02, 133.77, 131.29, 130.76, 129.69, 128.36, 127.36 127.12, 125.66, 125.25, 123.45, 119.87, 119.04, 117.51, 107.80, 106.58, 27.83, 24.78. HRMS: m/z = 441.1764[M − Br].

Synthesis of P1-P6:

Compound M1-M6 were copolymerized with acrylamide at the molar ratio of 1/50 (P1) was used as an example for demonstration. The polymer was prepared by copolymerization of the compound M1 (23.7 mg, 0.0597 mmol, 1 eq) and acrylamide (212.2 mg, 2.9852 mmol, 50 eq) by a radial polymerization with 2,2'-azobis(2-methylpropionitrile) (AIBN) (4 mg) as radical initiator at 65 °C under an argon atmosphere in DMF for 12 h. The resulting mixture was added into methanol to precipitate polymeric materials. Precipitation was repeatedly washed with methanol to give purified polymers. The actual copolymerization ratios of P1-P6 are determined to be 1:61, 1:57, 1:55, 1:63, 1:67, and 1:56, respectively.

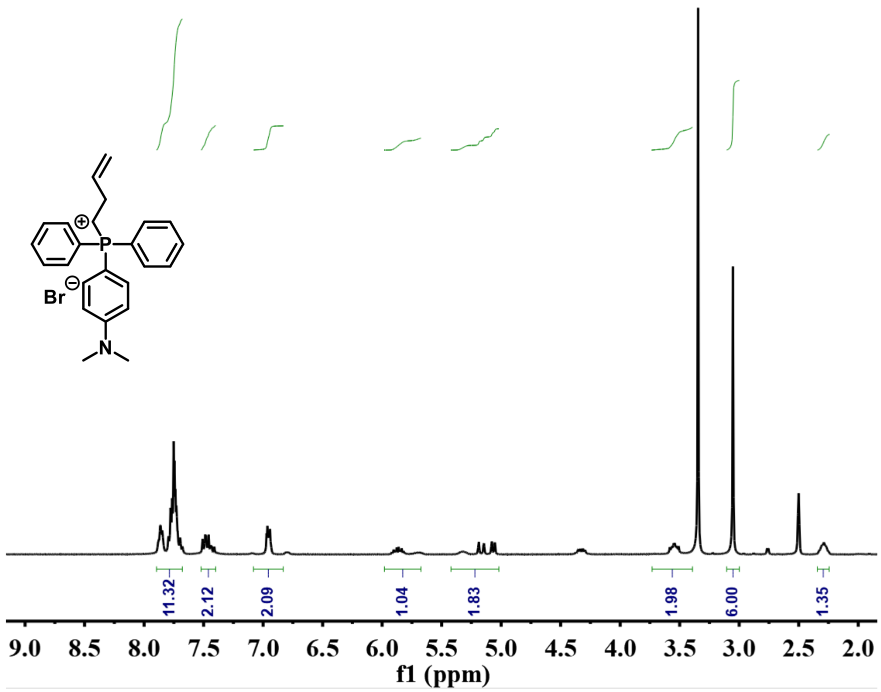

Supplementary Fig. 2 ^{1}H NMR spectrum of M1 in DMSO-d_6.

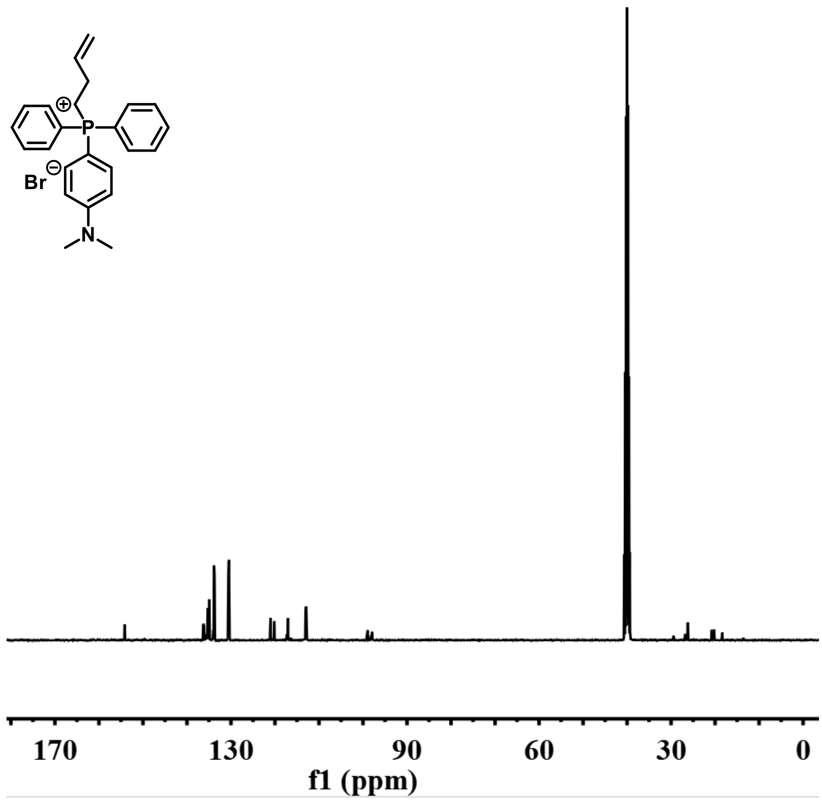

Supplementary Fig. 3 ^{13}C NMR spectrum of M1 in DMSO-d_6.

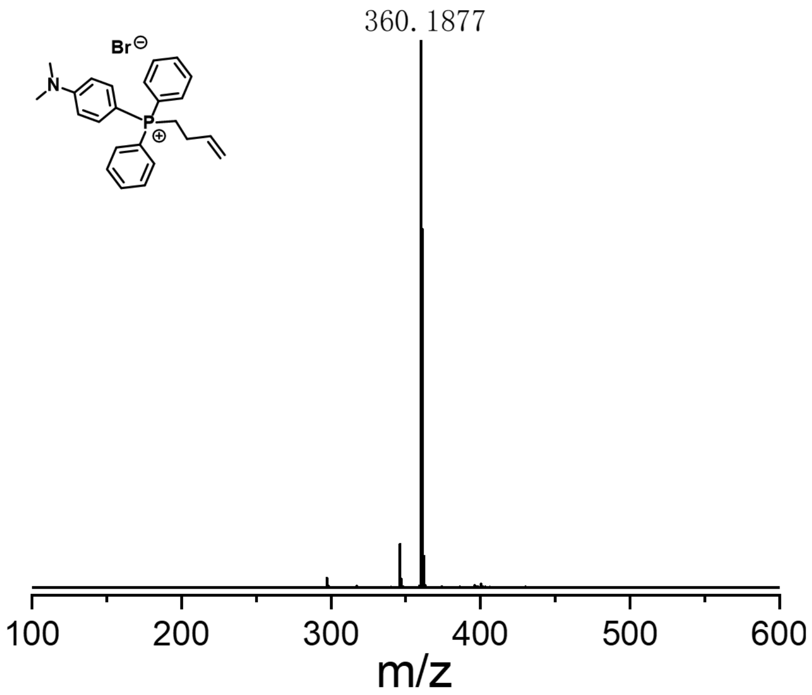

Supplementary Fig. 4 HRMS spectrum of M1.

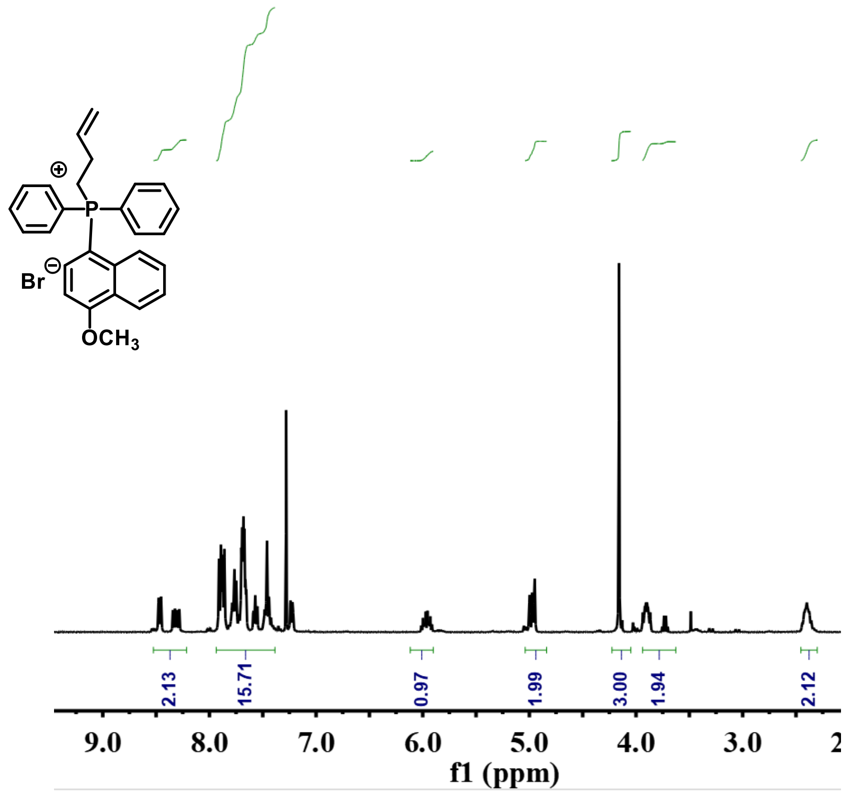

Supplementary Fig. 5 ^{1}H NMR spectrum of M2 in $CDCl_3$.

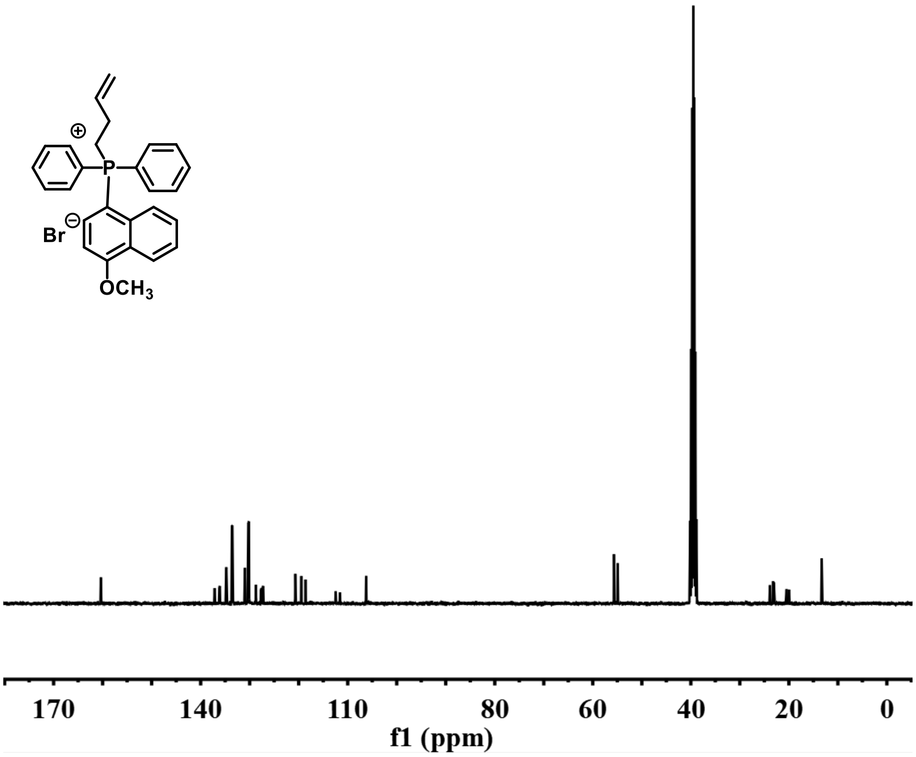

Supplementary Fig. 6 ^{13}C NMR spectrum of M2 in DMSO-d_6.

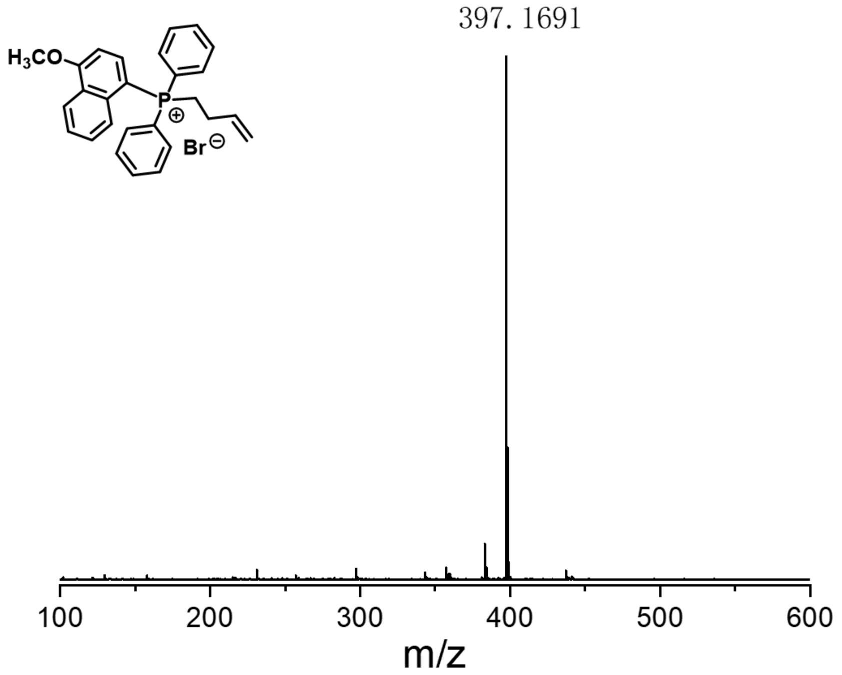

Supplementary Fig. 7 HRMS spectrum of M2.

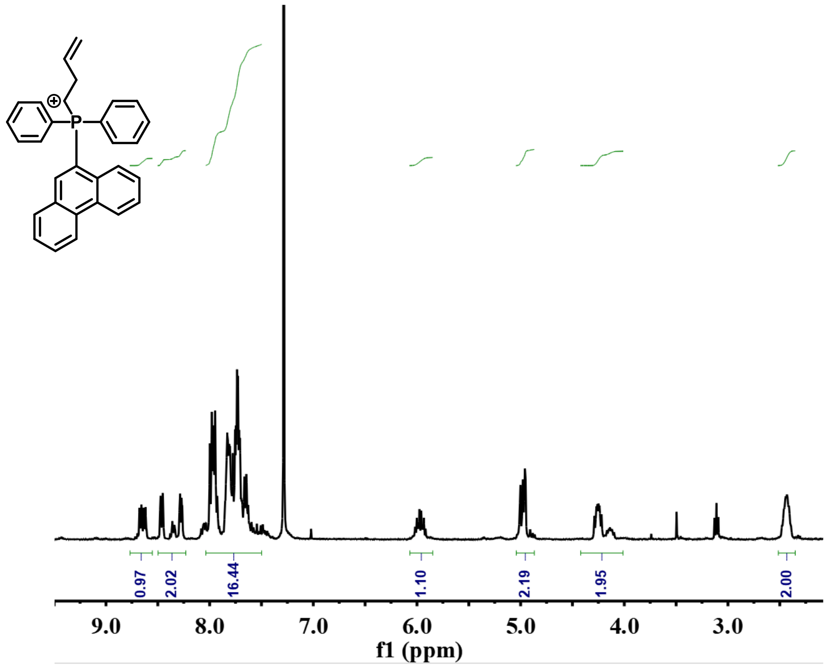

Supplementary Fig. 8 ^{1}H NMR spectrum of M3 in $CDCl_3$.

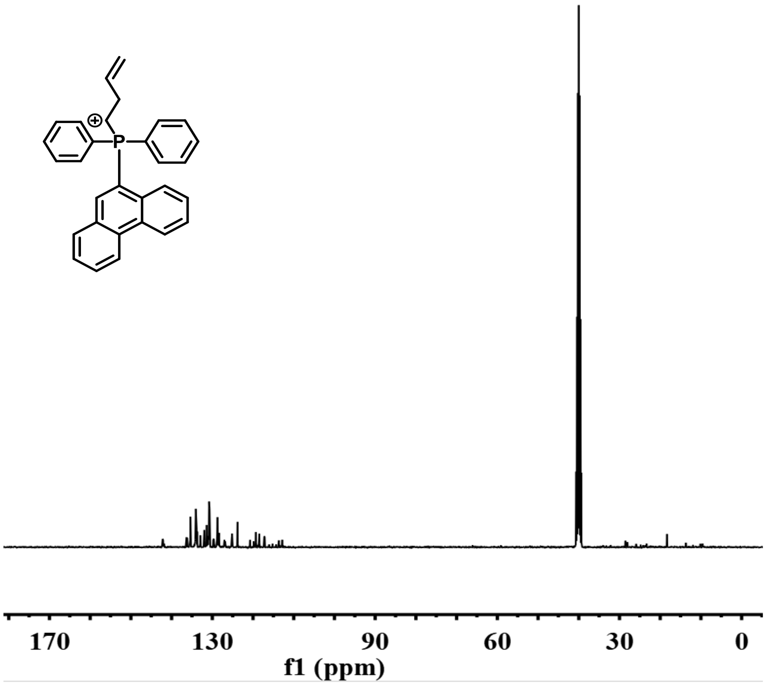

Supplementary Fig. 9 ^{13}C NMR spectrum of M3 in DMSO-d_6.

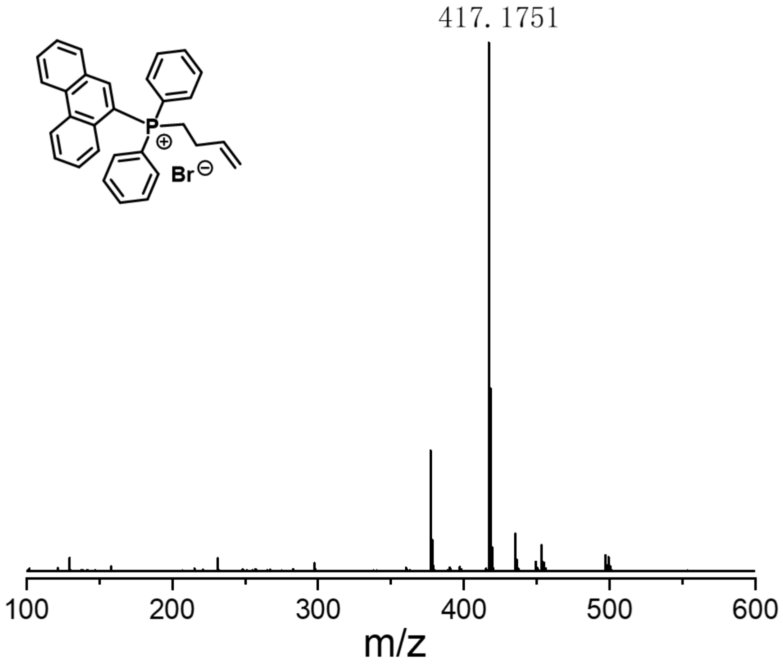

Supplementary Fig. 10 HRMS spectrum of M3.

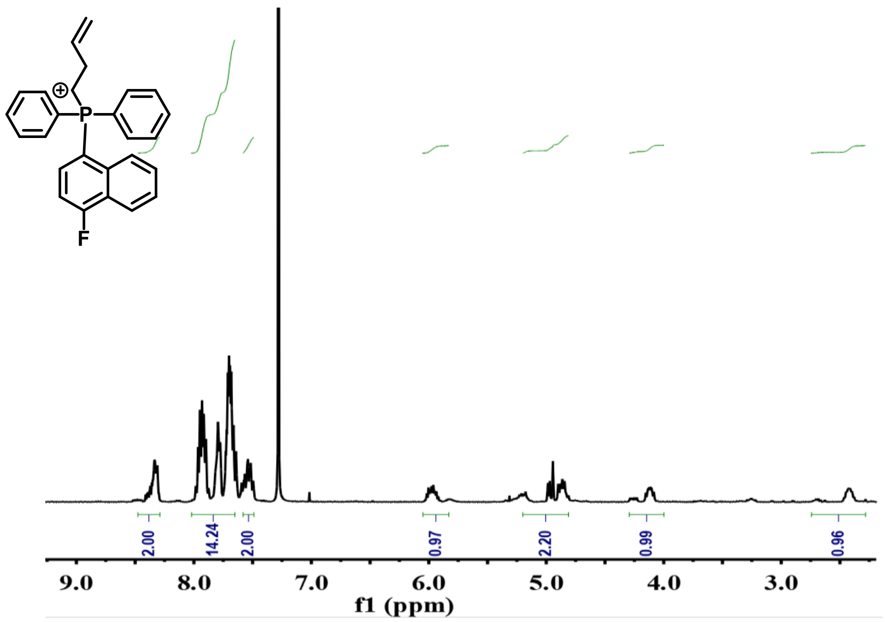

Supplementary Fig. 11 ^{1}H NMR spectrum of M4 in $CDCl_3$.

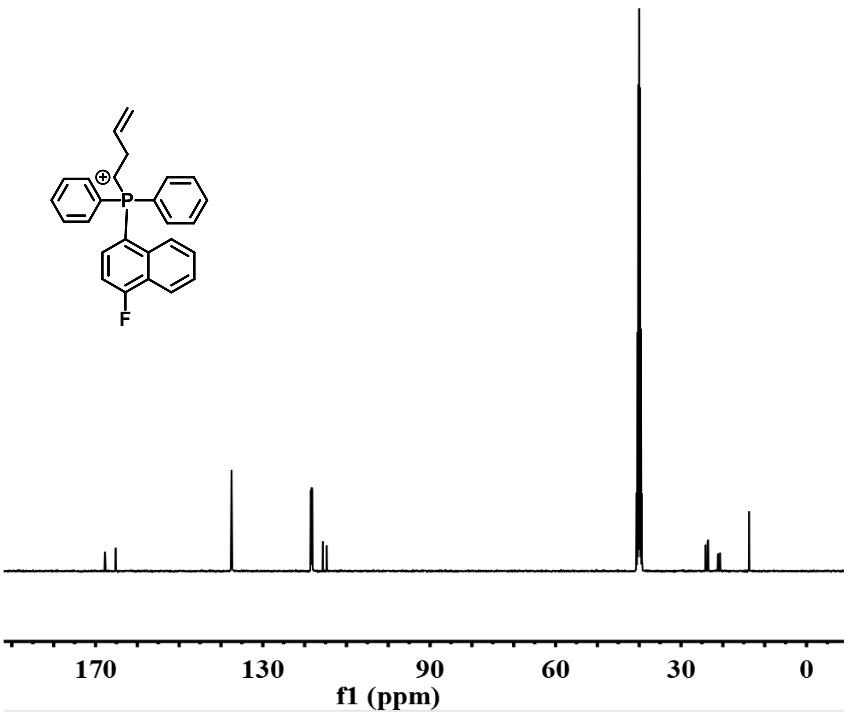

Supplementary Fig. 12 ^{13}C NMR spectrum of M4 in DMSO-d_6.

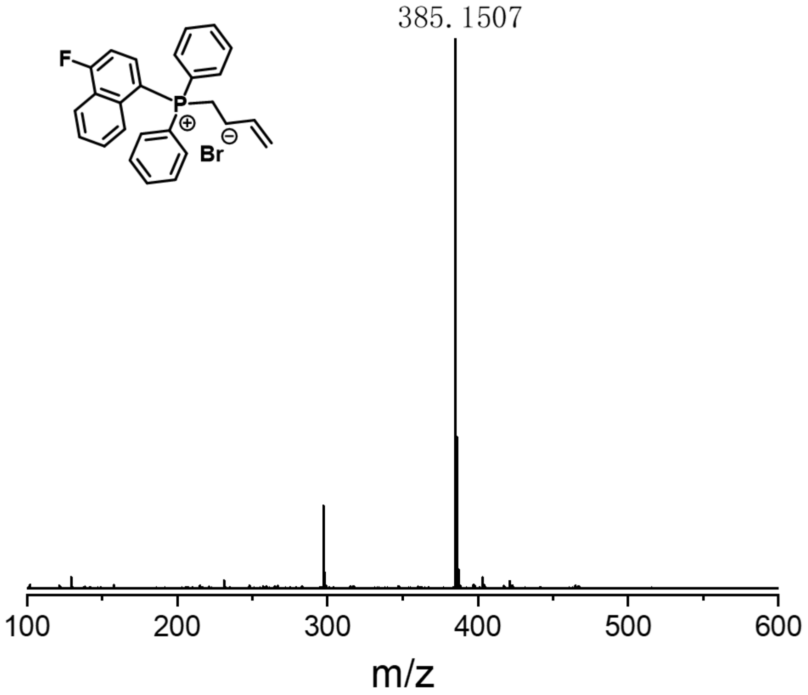

Supplementary Fig. 13 HRMS spectrum of M4.

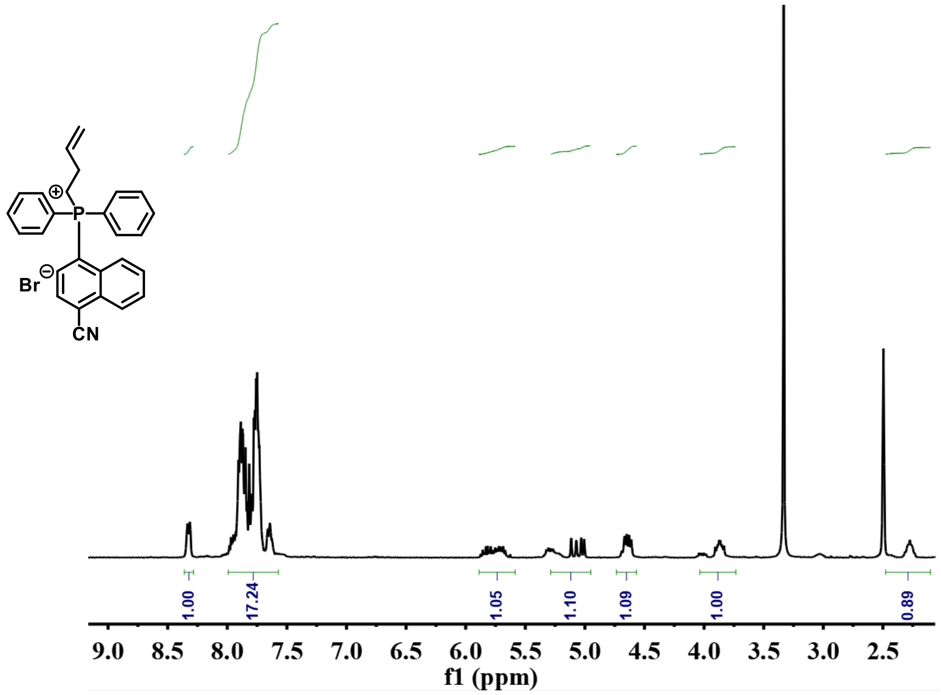

Supplementary Fig. 14 ^{1}H NMR spectrum of M5 in DMSO-d_6.

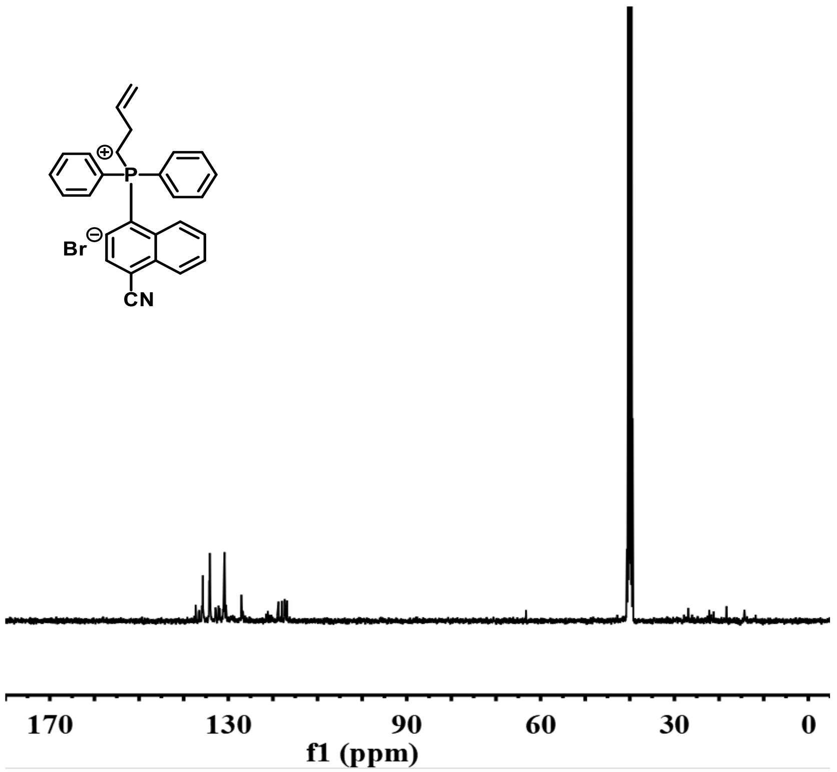

Supplementary Fig. 15 ^{13}C NMR spectrum of M5 in DMSO-d_6.

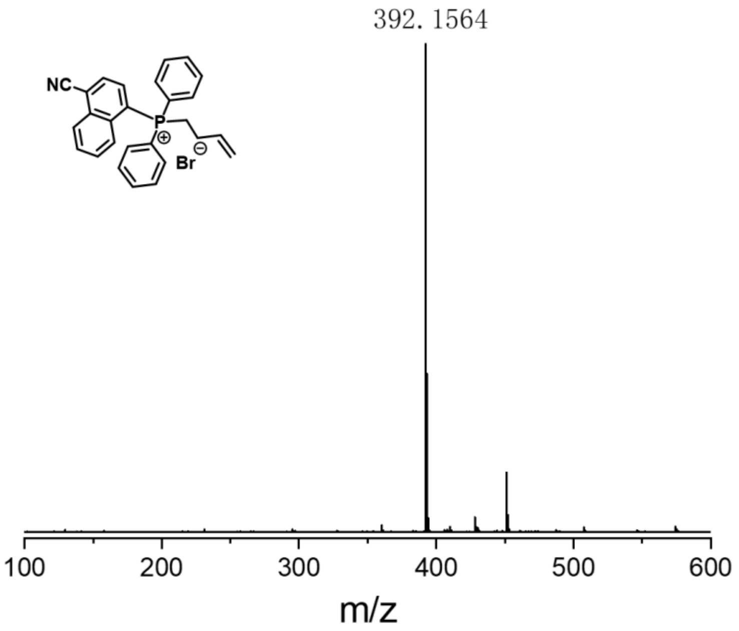

Supplementary Fig. 16 HRMS spectrum of M5.

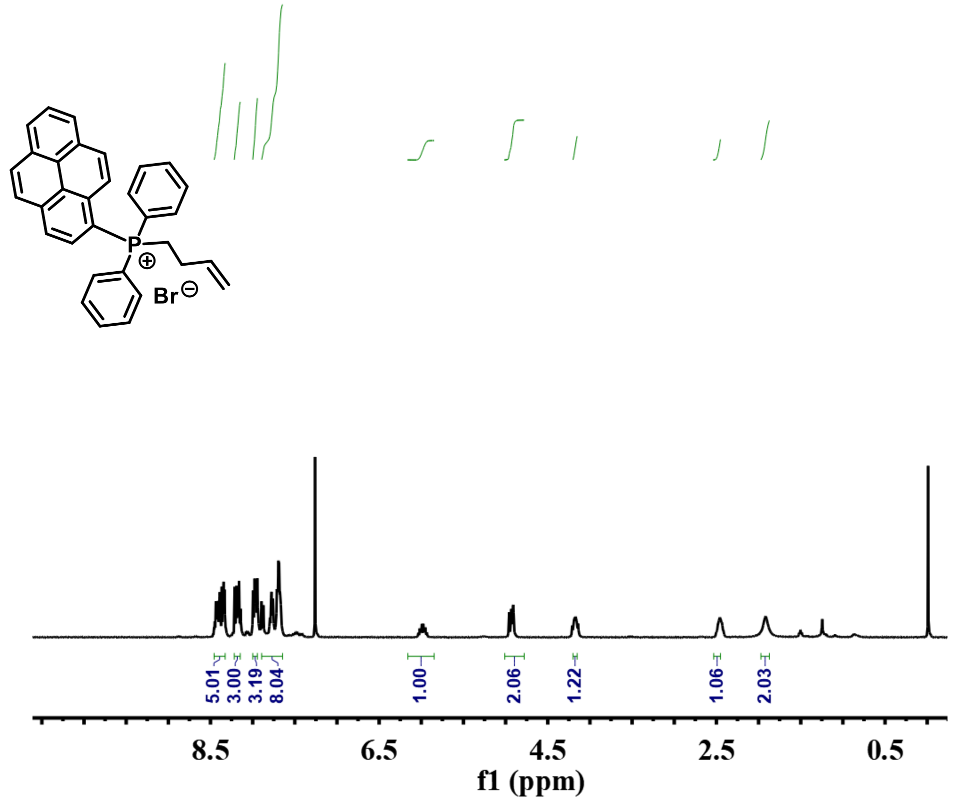

Supplementary Fig. 17 ^{1}H NMR spectrum of M6 in $CDCl_3$.

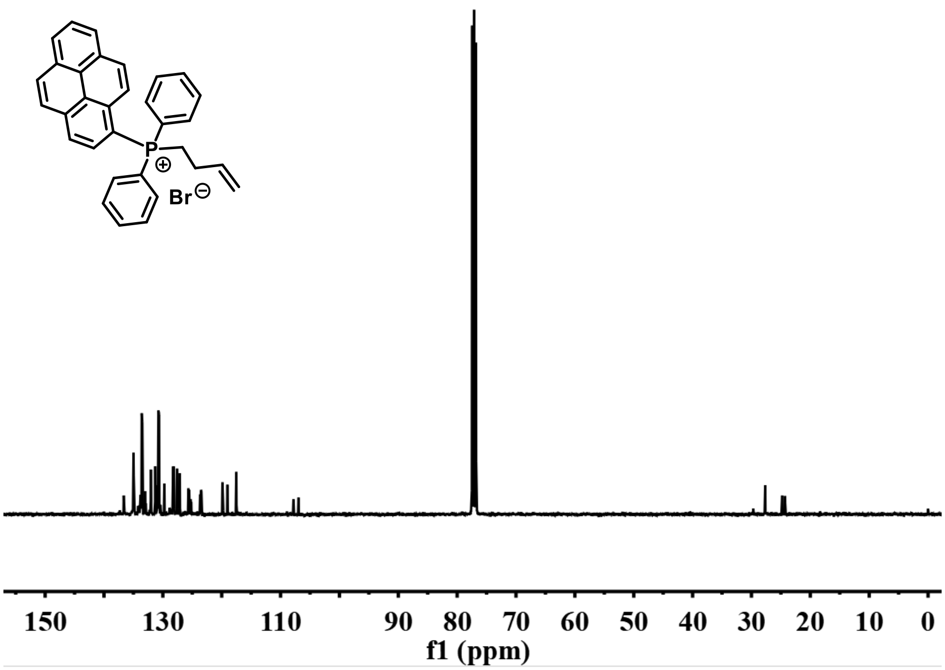

Supplementary Fig. 18 ^{13}C NMR spectrum of M6 in $CDCl_3$.

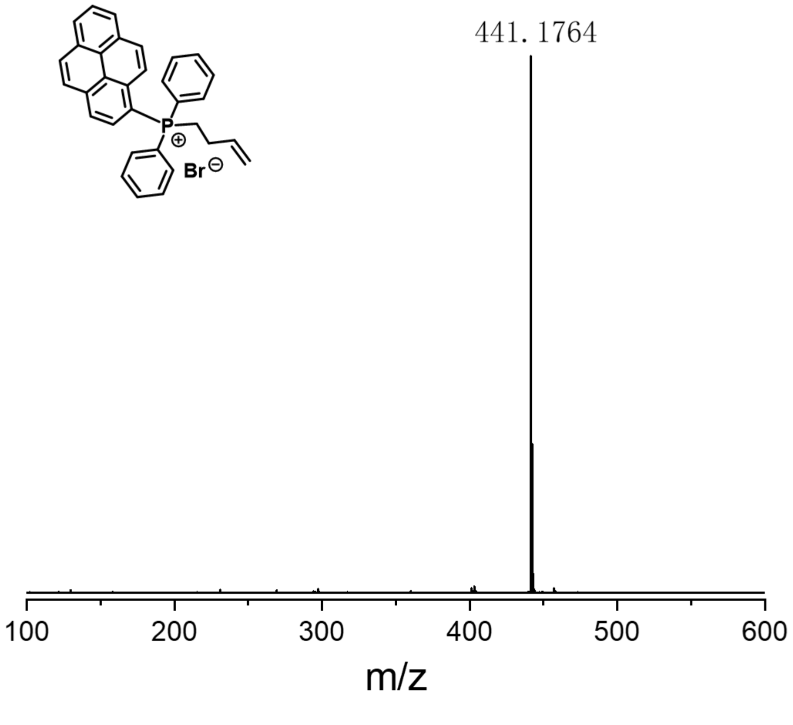

Supplementary Fig. 19 HRMS spectrum of M6.

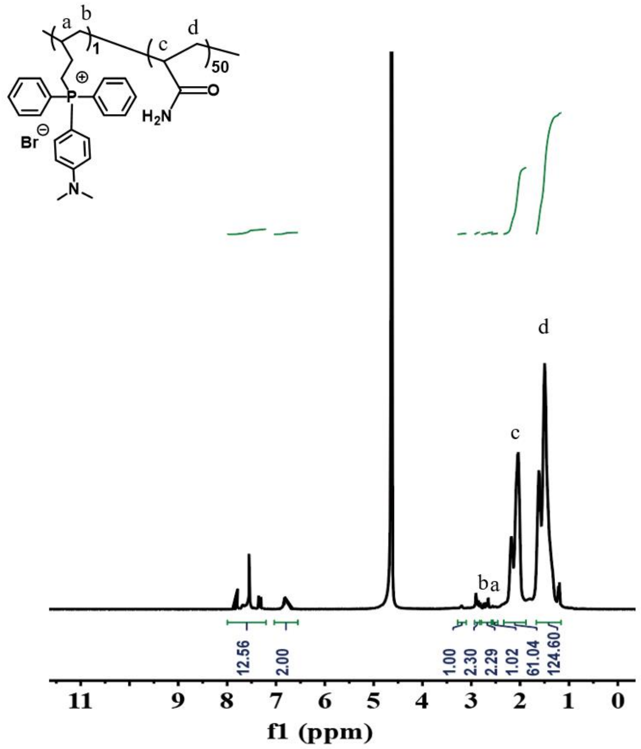

Supplementary Fig. 20 ^{1}H NMR spectrum of P1 in D_2O.

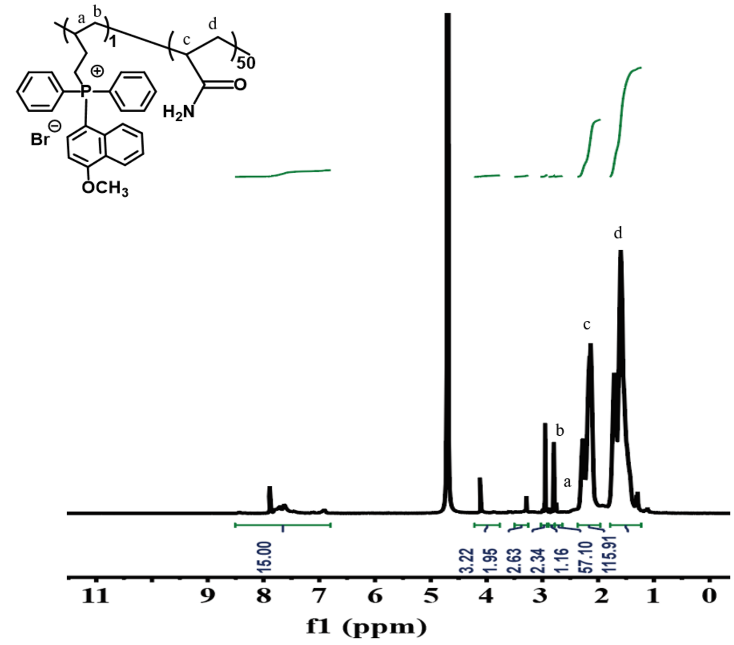

Supplementary Fig. 21 ^{1}H NMR spectrum of P2 in D_2O.

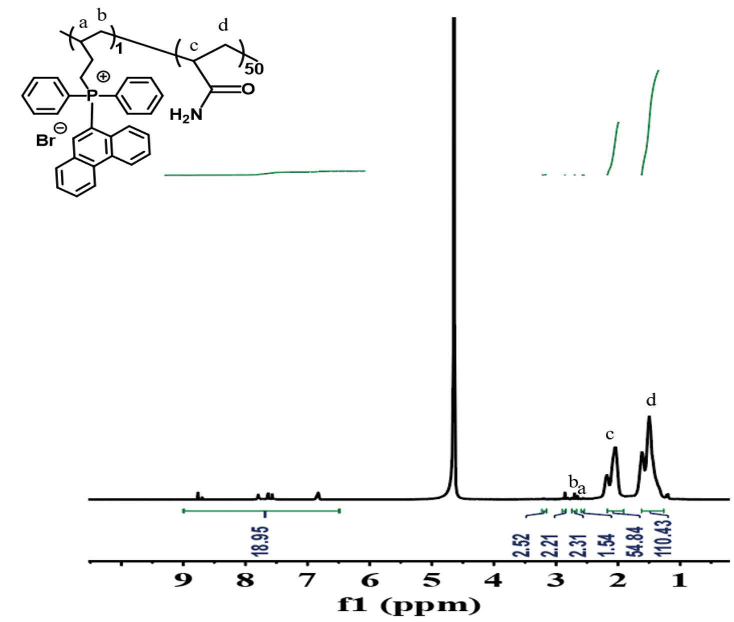

Supplementary Fig. 22 ^{1}H NMR spectrum of P3 in D_2O.

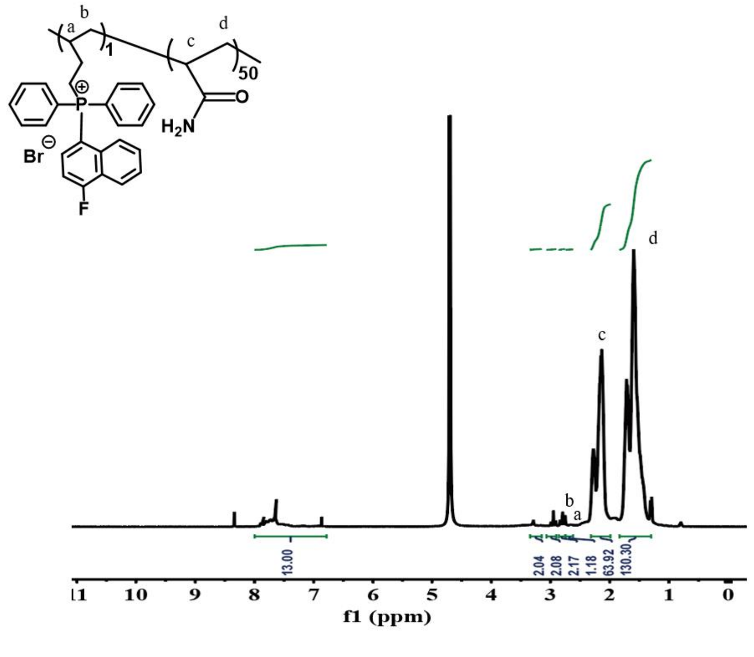

Supplementary Fig. 23 ^{1}H NMR spectrum of P4 in D_2O.

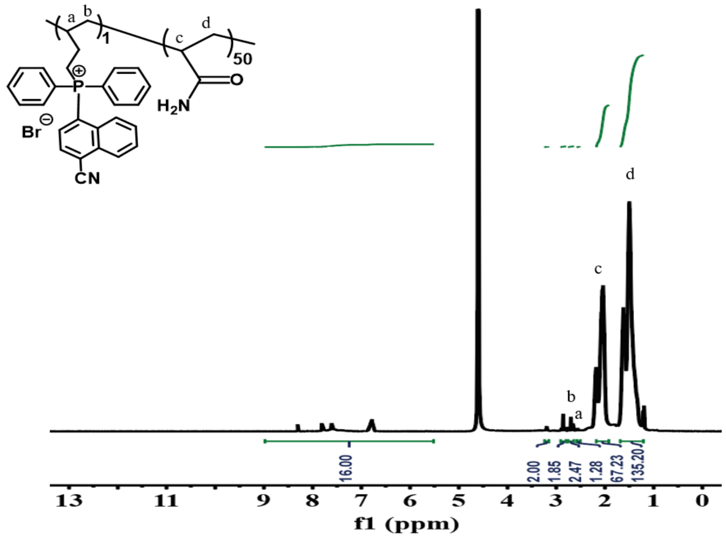

Supplementary Fig. 24 ^{1}H NMR spectrum of P5 in D_2O.

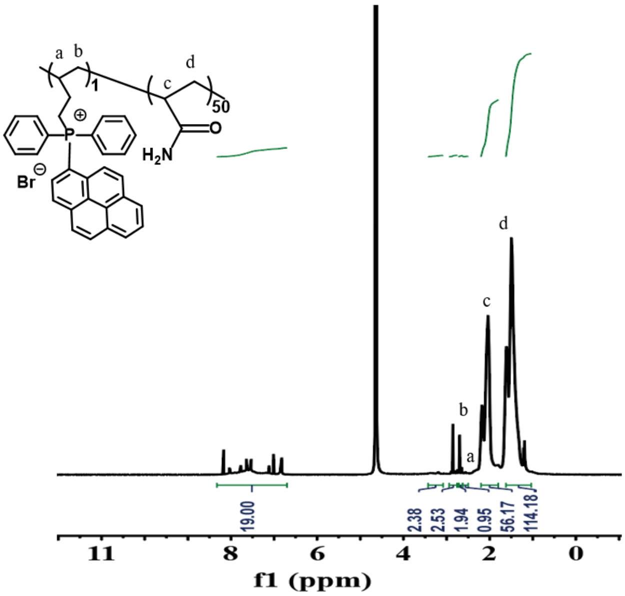

Supplementary Fig. 25 ^{1}H NMR spectrum of P6 in D_2O.

Supplementary Tab.S1 Characterizations of polymers. [a)]

Samples	Mw	Mn	PDI
P1(1/50)	28579	26771	1.0675
P2(1/50)	29674	18731	1.5842
P3(1/50)	38834	29877	1.2997
P4(1/50)	31324	24670	1.2697
P5(1/50)	33674	30185	1.1558
P6(1/50)	30989	28651	1.0816

a) Mw and Mn were determined by aqueous GPC

Fluorescence and phosphorescence QY calculation.

The method to calculate the fluorescence and phosphorescence quantum yields separately was performed according to previous literatures (*Angew. Chem. Int. Ed.* **2020**, *58*, 17451-17455; *Angew. Chem. Int. Ed.* **2020**, *59*, 16054-16060). The phosphorescence bands could be obtained in their delayed emission spectrum. According to the structure of phosphorescence bands, the fluorescence and phosphorescence emission bands could be separated in steady state emission spectra. The ratio for fluorescence and phosphorescence quantum yields could be calculated with areas of separated fluorescence and phosphorescence bands. Thus, the fluorescence and phosphorescence quantum yields could be obtained with their total luminescence quantum yields and the ratio for the two relative quantum yields. The calculation method was added in the revised supplementary information.

Photoluminescence quantum efficiency was determined by using Edinburgh FLS980 spectrometer with the integrating sphere (142 mm in diameter) under ambient condition, the fluorescence and phosphorescence quantum efficiency (Φ_F and Φ_P) were calculated through the following formulas:

$$\emptyset_P = \emptyset_E \times \frac{A_P}{A_E} \qquad (1)$$

$$\emptyset_F = \emptyset_E - \emptyset_P \qquad (2)$$

where Φ_E refers to the measured total emission quantum efficiency, AP and AE refer to the integral areas of phosphorescence and photoluminescence components in photoluminescence spectra, respectively.

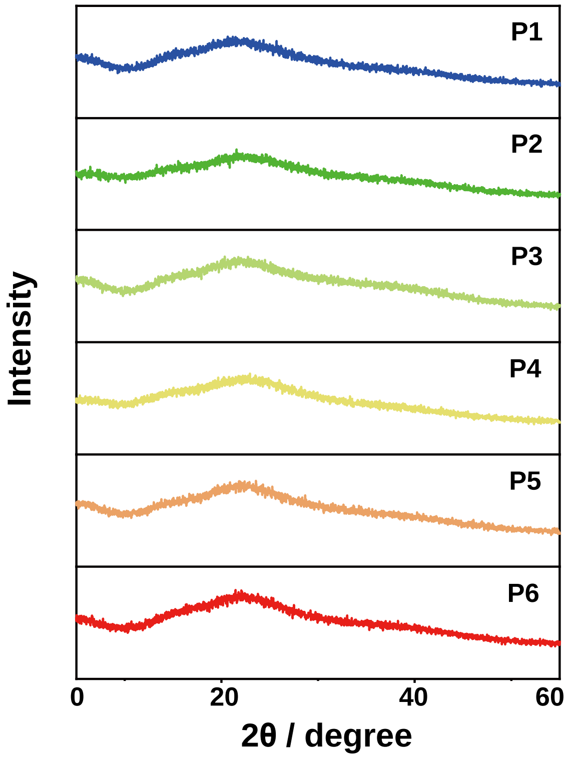

Supplementary Fig. 26 PXRD pattern of P1-P6

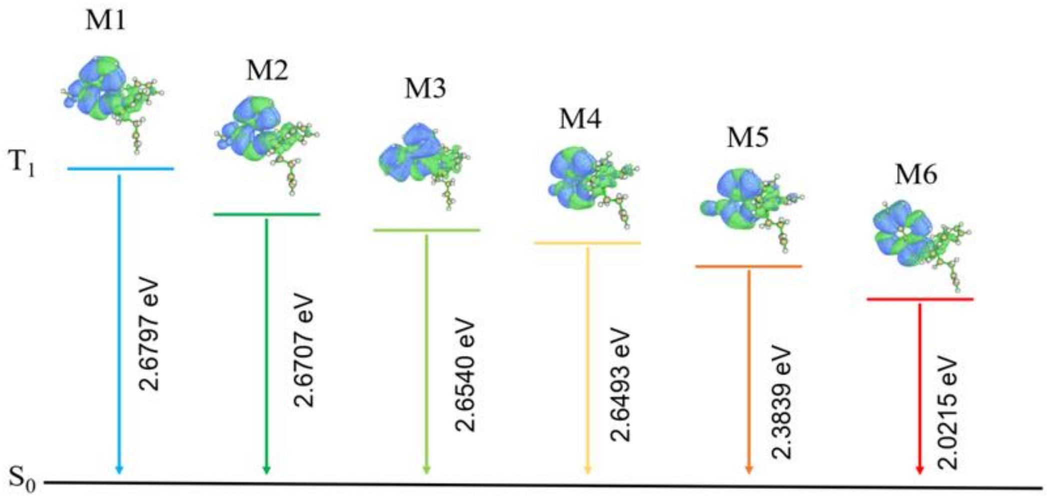

Supplementary Fig. 27 The frontier molecular orbitals of the lowest excited triplet states for various monomers M1-M6, as calculated using the TDDFT with B3LYP functional and def2-SVP basis set.

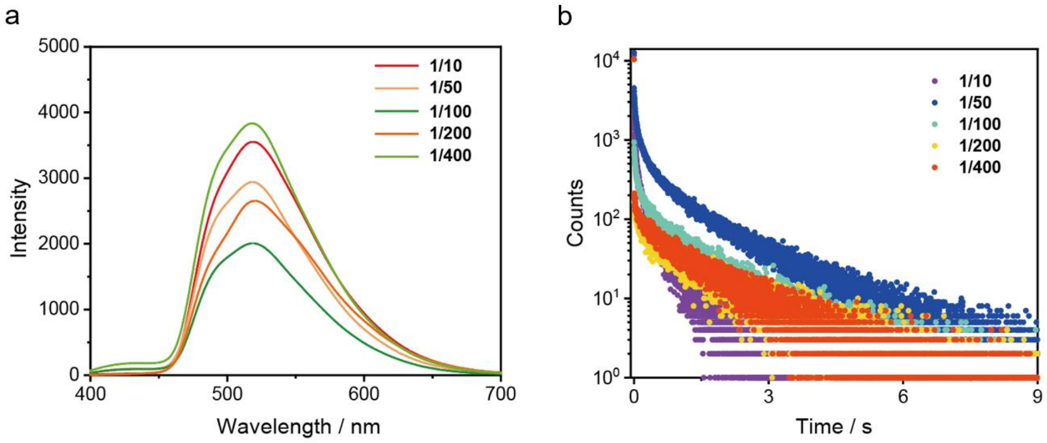

Supplementary Fig. 28 a The phosphorescence spectra of P3 with different ratios (1/10-1/400) under 365 nm excitation. **b** Emission lifetime decay curves of P3 (1/10-1/400) in the solid state under 365 nm excitation.

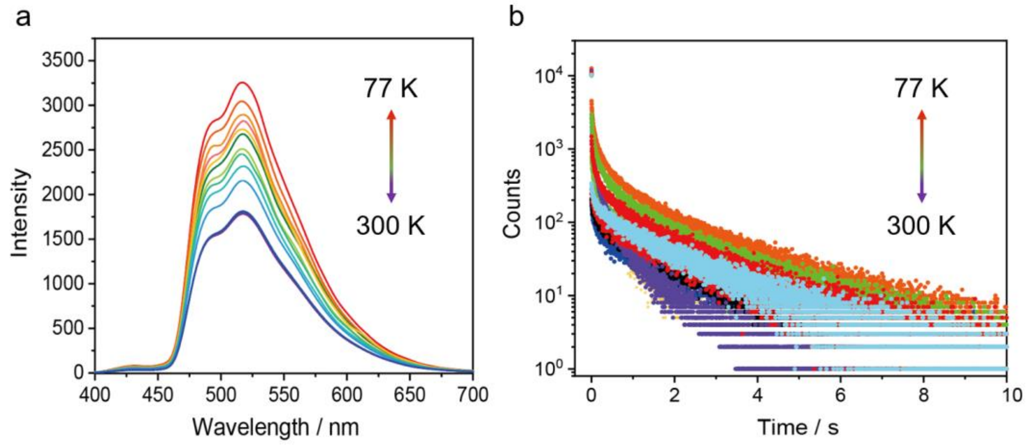

Supplementary Fig. 29 a Temperature dependence (77 K-300 K) phosphorescence spectra of P3 (1/50) under 365 nm excitation. **b** Temperature dependence (77 K-300 K) emission lifetime decay curves of P3 (1/50) in the solid state under 365 nm excitation.

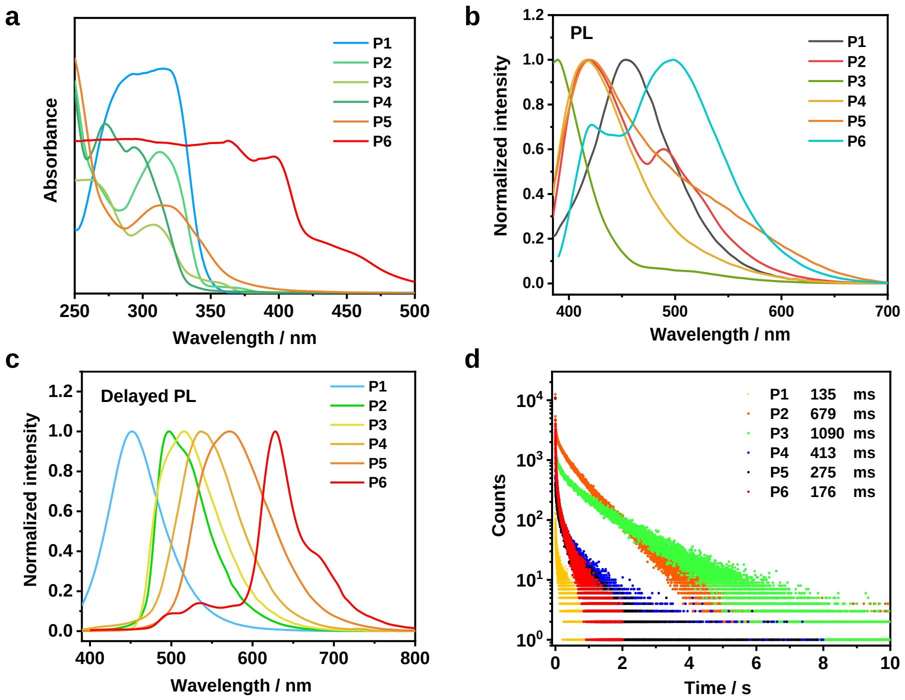

Supplementary Fig. 30 Photophysical properties of P1-P6. a The UV absorption spectra of polymers P1-P6 at room temperature. **b** The PL spectra of polymers P1-P6 in the solid state at 365nm excitation wavelengths. **c** The decay PL spectra of polymers P1-P6 in the solid state at 365nm excitation wavelengths. **d** Lifetime decay curves of P1-P6 under ambient condition.

Supplementary Tab.2 Photophysical properties of P1-P6 under ambient conditions excitation wavelength.

Compounds	Ex(max)	Lifetime					Φ[%]	Φ_P[%]
		τ_1(ms)	A_1(%)	τ_2(ms)	A_2(%)	τ_{ave}(ms)		
P1	340 nm	15.89	34	246.08	66	167.40	18.89	1.83
P2	365 nm	227.24	18	768.75	82	679.28	12.64	2.98
P3	320 nm	285.39	16	1301.94	84	1139.29	27.17	6.58
P4	340 nm	81.73	19	761.20	81	632.10	21.53	1.51
P5	330 nm	11.41	27	466.63	73	343.72	15.45	1.64
P6	370 nm	17.41	14	267.92	86	231.50	22.86	2.61

Supplementary Discussion

Mechanism diagram for grafting of PDMS and PVA

Chemical modification has two main methods, including plasma grafting and surface modification. PVA is widely used to blend with other polymers to improve their hydrophilicity and biocompatibility (*Surf. Coat. Tech.* **2012**, 206, 2161; *Sci. Rep.* **2018**, 8, 16038). In our work, we employ the plasma grafting method to improve the hydrophilicity of PDMS. As shown in Figure R1, with the increase of 365 nm irradiation time, PDMS generates more active groups and active sites to bind more PVA, which raise the hydrophilicity of PDMS. Next, P3 polymers are embedded into PDMS-PVA chains through hydrogen bonding, van-der-Walls, ion-dipole, and dipole-dipole interactions. Finally, the mixture was transferred into a fluoridated mold, and elastomer films were obtained after cooling.

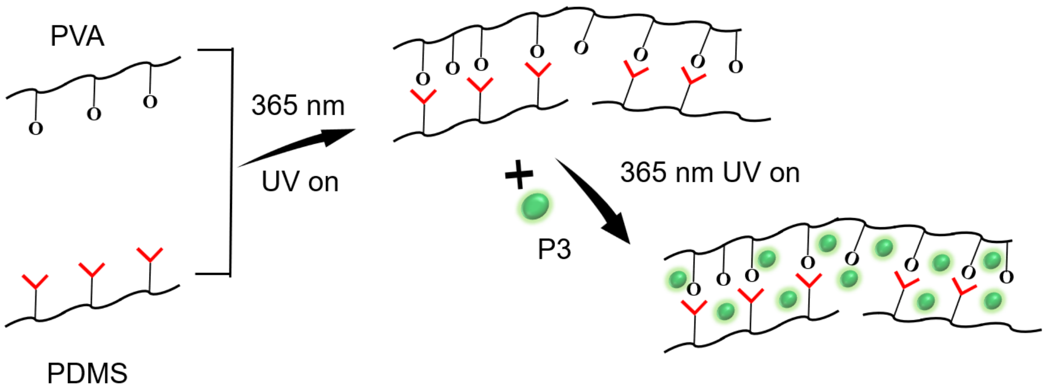

Supplementary Fig. 31 Mechanism diagram for grafting of PDMS and PVA.

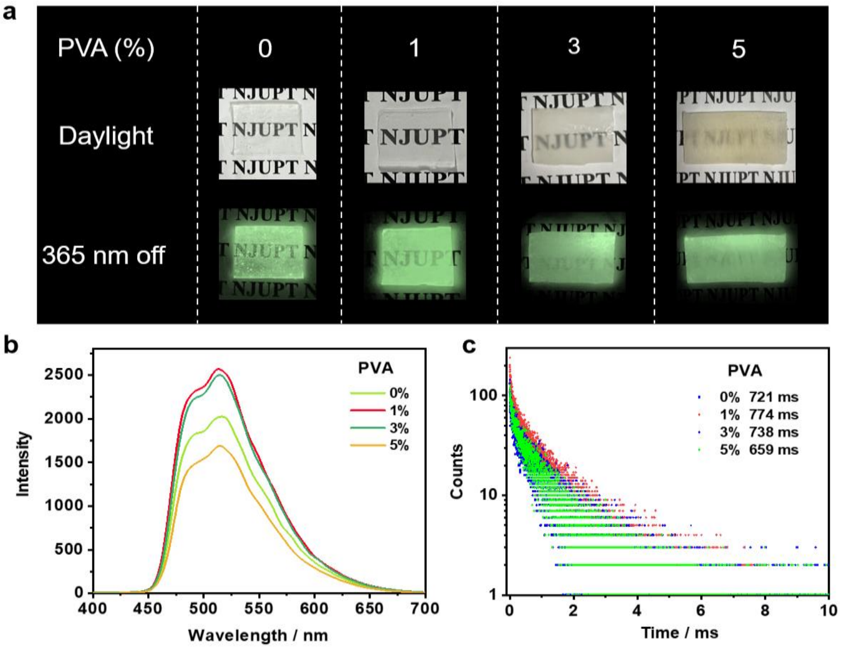

Supplementary Fig. 32 Photophysical properties of different amounts of PVA elastomer films. a The Photos of different amounts of PVA elastomer films in daylight and 365nm UV light off. **b** The decay PL spectra of different amounts of PVA elastomer films at 365nm excitation wavelengths. **c** Lifetime decay curves of different amounts of PVA elastomer films at 365nm excitation wavelengths.

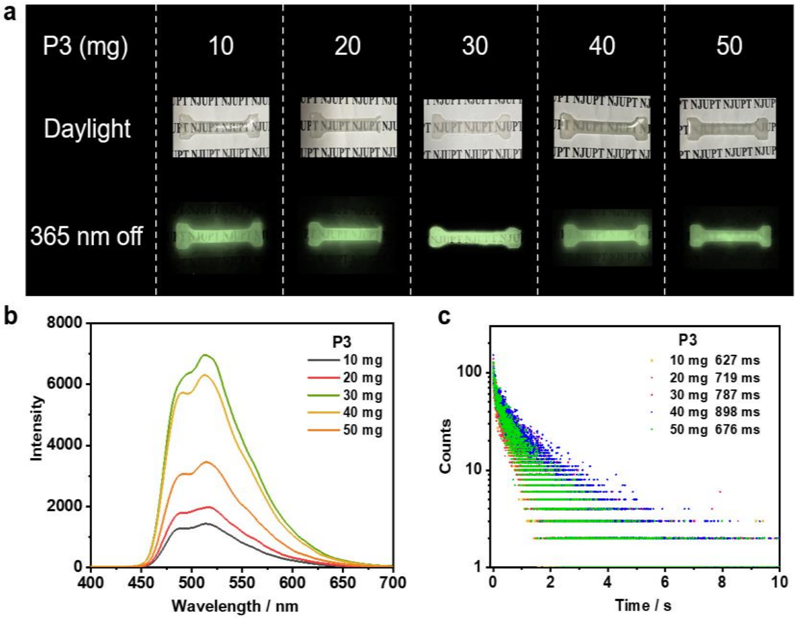

Supplementary Fig. 33 Photophysical properties of different amounts of P3 elastomer films. a The Photos of different amounts of P3 elastomer films in daylight and 365nm UV light off. **b** The decay PL spectra of different amounts of P3 elastomer films at 365nm excitation wavelengths. **c** Lifetime decay curves of different amounts of P3 elastomer films at 365nm excitation wavelengths.

Supplementary Tab.3 Photophysical properties of E1-E6 under 365nm excitation wavelength.

Compounds	Ex(nm)	Lifetime					Φ[%]	Φ_P[%]
		τ_1(ms)	A_1(%)	τ_2(ms)	A_2(%)	τ_{ave}(ms)		
P1	365	17.98	25	104.02	75	82.49	13.98	1.35
P2		27.24	12	547.74	88	485.28	12.64	2.17
P3		87.38	17	936.39	83	792.06	14.41	4.04
P4		31.88	20	273.20	80	224.94	21.03	2.08
P5		7.64	33	211.74	67	144.37	14.96	1.45
P6		17.41	14	109.30	86	137.43	16.86	1.57

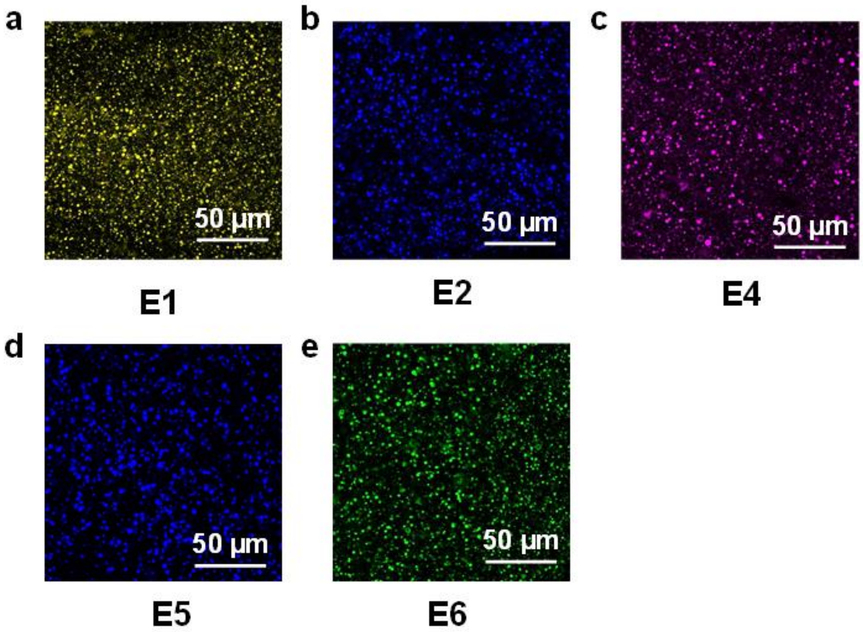

Supplementary Fig. 34 Confocal images of E1-E6. a-e The Confocal images of E1-E6 films at 405 nm excitation wavelengths.

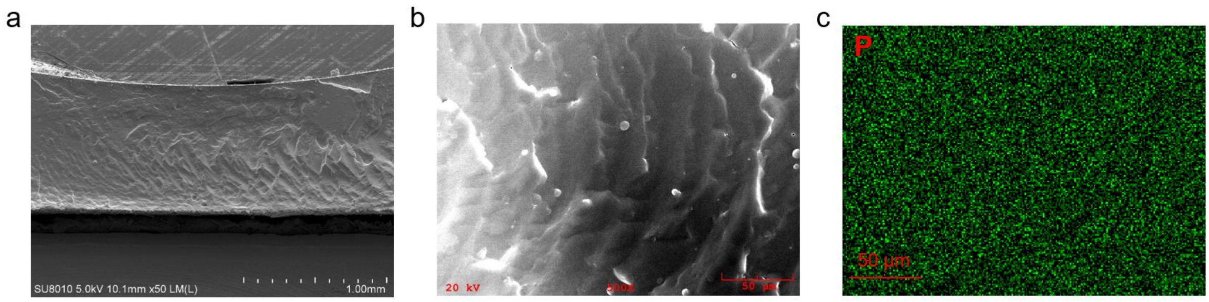

Supplementary Fig. 35 The SEM properties of E3. a, b SEM image of E3 fracture section. c EDS images of E3 for P.

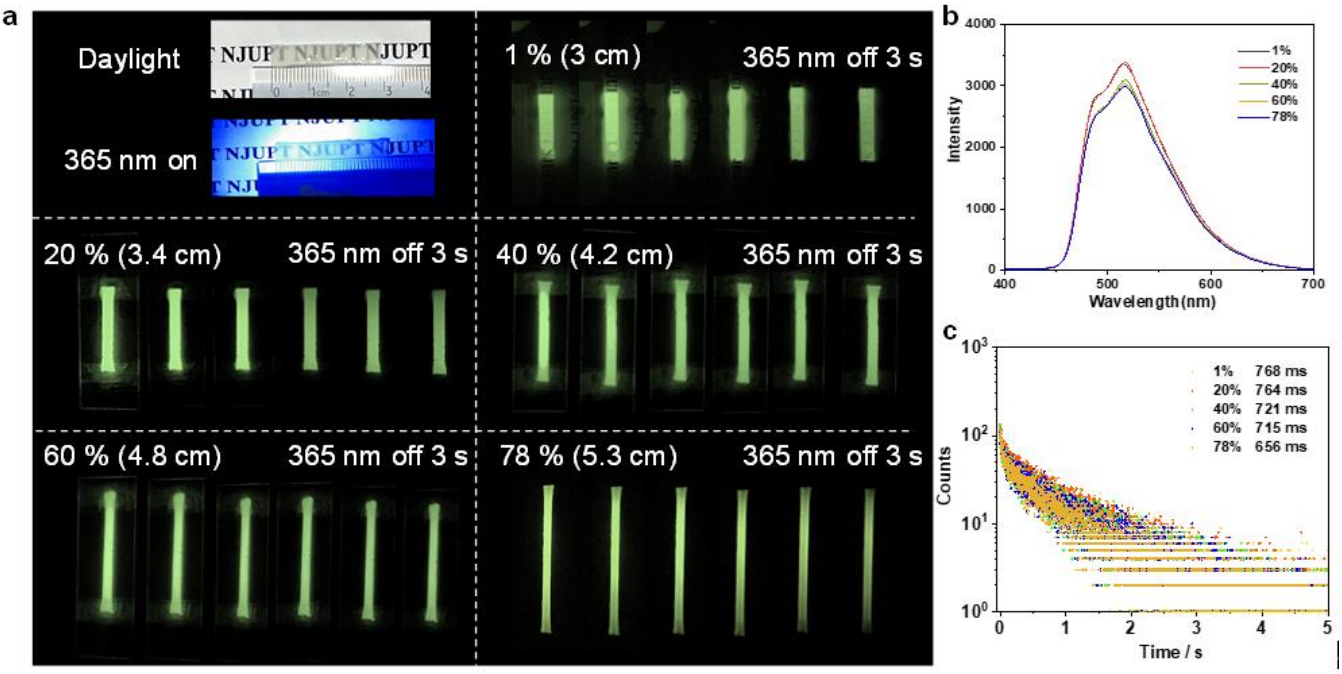

Supplementary Fig. 36 The photophysical properties of E3 in the different stretched states. a The Photos of different stretched states of E3 under 365nm UV light off (1-78 %). **b** The decay PL spectra of different amounts of E3 at 365nm excitation wavelengths (1-78 %). **c** Lifetime decay curves of different stretched states of E3 at 365nm excitation wavelengths (1-78 %).

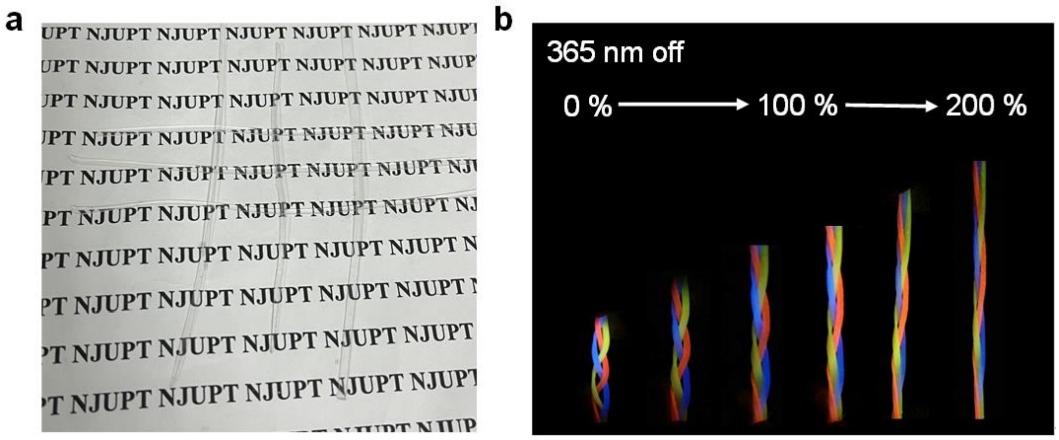

Supplementary Fig. 37 The persistent RTP fibers prepared by E1, E3, and E6. a Transparent RTP fibers under the daylight. **b** The Photos of different stretched states of RTP fibers under 365nm UV light off.

Supplementary Reference

1. Lei, Y.X. et al. Wide-range color-tunable organic phosphorescence materials for printable and writable security inks. *Angew. Chem. Int. Ed.* **59**, 16054-16060 (2020).
2. Yang Z. et al. Boosting the quantum efficiency of ultralong organic phosphorescence up to 52% via intramolecular halogen bonding. *Angew. Chem. Int. Ed.* **59**, 17451-17455 (2020).
3. Li, J. Y. et al. Chemical modification on top of nanotopography to enhance surface properties of PDMS. *Surf. Coat. Tech.* **206**, 2161 (2012).
4. Perween, S. et al. PVA-PDMS-stearic acid composite nanofibrous mats with improved mechanical behavior for selective filtering applications. *Sci. Rep.* **8**, 16038 (2018).
