# Supplementary material for: Full-color persistent room temperature phosphorescent elastomers with robust optical properties
Source: Nat Commun. 2023 Aug 10;14:4839. doi: 10.1038/s41467-023-40193-1 (PMC10415293; doi:10.1038/s41467-023-40193-1)
Supplement: Supplementary file 3 — Description of Additional Supplementary Files [file 41467_2023_40193_MOESM3_ESM.pdf]

Description of Additional Supplementary Files

File Name: Supplementary Movie 1

Description: The E3 film exhibits a green afterglow emission upon the removal of 365 nm excitation.

File Name: Supplementary Movie 2

Description: The stretched E3 film exhibits a green afterglow emission upon the removal of 365 nm excitation.

File Name: Supplementary Movie 3

Description: The E3 film shows s green afterglow emission during multiple stretch-release cycles, after the removal of 365 nm excitation.

File Name: Supplementary Movie 4

Description: The stretched E1, E4, and E6 films exhibit blue, yellow, and red afterglow emissions upon the removal of 365 nm excitation.
